# Utilizing Chemical Genomics to Identify Cytochrome *b* as a Novel Drug Target for Chagas Disease

**DOI:** 10.1371/journal.ppat.1005058

**Published:** 2015-07-17

**Authors:** Shilpi Khare, Steven L. Roach, S. Whitney Barnes, Dominic Hoepfner, John R. Walker, Arnab K. Chatterjee, R. Jeffrey Neitz, Michelle R. Arkin, Case W. McNamara, Jaime Ballard, Yin Lai, Yue Fu, Valentina Molteni, Vince Yeh, James H. McKerrow, Richard J. Glynne, Frantisek Supek

**Affiliations:** 1 Department of Genetics and Neglected Diseases, Genomics Institute of the Novartis Research Foundation, San Diego, California, United States of America; 2 Department of Medicinal Chemistry, Genomics Institute of the Novartis Research Foundation, San Diego, California, United States of America; 3 Novartis Institutes for BioMedical Research, Novartis Campus, Basel, Switzerland; 4 Small Molecule Discovery Center and Department of Pharmaceutical Chemistry, University of California, San Francisco, San Francisco, California, United States of America; 5 Skaggs School of Pharmacy and Pharmaceutical Sciences, University of California, San Diego, La Jolla, California, United States of America; University of Texas Southwestern Medical Center, UNITED STATES

## Abstract

Unbiased phenotypic screens enable identification of small molecules that inhibit pathogen growth by unanticipated mechanisms. These small molecules can be used as starting points for drug discovery programs that target such mechanisms. A major challenge of the approach is the identification of the cellular targets. Here we report GNF7686, a small molecule inhibitor of *Trypanosoma cruzi*, the causative agent of Chagas disease, and identification of cytochrome *b* as its target. Following discovery of GNF7686 in a parasite growth inhibition high throughput screen, we were able to evolve a GNF7686-resistant culture of *T*. *cruzi* epimastigotes. Clones from this culture bore a mutation coding for a substitution of leucine by phenylalanine at amino acid position 197 in cytochrome *b*. Cytochrome *b* is a component of complex III (cytochrome *bc_1_*) in the mitochondrial electron transport chain and catalyzes the transfer of electrons from ubiquinol to cytochrome *c* by a mechanism that utilizes two distinct catalytic sites, Q_N_ and Q_P_. The L197F mutation is located in the Q_N_ site and confers resistance to GNF7686 in both parasite cell growth and biochemical cytochrome *b* assays. Additionally, the mutant cytochrome *b* confers resistance to antimycin A, another Q_N_ site inhibitor, but not to strobilurin or myxothiazol, which target the Q_P_ site. GNF7686 represents a promising starting point for Chagas disease drug discovery as it potently inhibits growth of intracellular *T*. *cruzi* amastigotes with a half maximal effective concentration (EC_50_) of 0.15 µM, and is highly specific for *T*. *cruzi* cytochrome *b*. No effect on the mammalian respiratory chain or mammalian cell proliferation was observed with up to 25 µM of GNF7686. Our approach, which combines *T*. *cruzi* chemical genetics with biochemical target validation, can be broadly applied to the discovery of additional novel drug targets and drug leads for Chagas disease.

## Author Summary

Chagas Disease, or American trypanosomiasis, is caused by the kinetoplastid protozoan *Trypanosoma cruzi* and is primarily transmitted to a mammalian host via a triatomine insect vector (the “kissing bug”) infected with *T*. *cruzi* parasites. Although discovered in 1909 by the physician Dr. Carlos Chagas, the disease gained recognition by the public health community only following a major outbreak in Brazil during the 1960s. Approximately eight million people (primarily in Central and South America) are infected with *T*. *cruzi* and cases are becoming more widespread due to migration out of the endemic regions. Current treatment options have severe problems with toxicity, limited efficacy, and long administration. Hence, discovery of new drugs for treatment of Chagas disease has become of prime interest to the biomedical research community. In this study, we report identification of a potent inhibitor of *T*. *cruzi* growth and use a chemical genetics-based approach to elucidate the associated mechanism of action. We found that this compound, GNF7686, targets cytochrome *b*, a component of the mitochondrial electron transport chain crucial for ATP generation. Our study provides new insights into the use of phenotypic screening to identify novel targets for kinetoplastid drug discovery.

## Introduction

Chagas disease, or American trypanosomiasis, is a neglected disease caused by the kinetoplastid protozoan *Trypanosoma cruzi* (*T*. *cruzi*). Endemic to Latin America, Chagas disease is increasingly globalized due to population migration from endemic regions into developed countries, and the U.S. in particular. About eight million people are estimated to harbor the infection with 40,000 new cases added annually [1, 2].

In the 100+ years that have passed since the first description of Chagas disease by Carlos Chagas, only two drugs have been developed to treat this infection: nifurtimox and benznidazole [2, 3]. While both these drugs can clear *T*. *cruzi* from the infected mammalian hosts, they are both inadequate to address the medical need of millions of patients chronically infected today. The drug shortcomings include toxicity, prolonged treatment time, and high rate of treatment failure [2, 3].

*T*. *cruzi* is transmitted to mammalian hosts primarily via hematophagous triatomine bugs [4]. While in the vector insect, *T*. *cruzi* cells propagate as flagellated epimastigotes that transform into non-dividing infective trypomastigotes. As the *T*. *cruzi*-infected bug takes a blood meal from a host, it deposits motile *T*. *cruzi* trypomastigotes near the wound site. Following invasion of host cells in the bite wound or at mucous membranes, intracellular trypomastigotes undergo a morphological transformation into amastigotes and start to replicate [1, 4, 5]. After completing several rounds of intracellular division, the amastigotes transform into trypomastigotes that then leave the infected cell and initiate a new cycle of infection.

The acute phase of Chagas disease is often asymptomatic, characterized by readily detectable parasitemia, and usually resolves within a few weeks through control of parasite proliferation by the adaptive immune system [4]. In the chronic stage, infected individuals rarely display symptoms or evidence of the disease for decades. However, ~30% of these patients eventually go on to develop a severe cardiomyopathy and ~10% of patients progress with gastrointestinal complications [1, 4].

New drug discovery for Chagas disease is hampered by very limited number of validated *T*. *cruzi* drug targets. Drug discovery efforts have focused on the trypanosome ergosterol biosynthesis pathway and cruzain, a *T*. *cruzi* cysteine protease [6]. During the last decade, much attention has been paid to inhibitors of sterol 14 α-demethylase (CYP51), an essential enzyme in the ergosterol biosynthesis pathway [6, 7]. To a large degree, this interest has been fueled by the availability of drugs targeting fungal sterol 14 α-demethylase, such as posaconazole or ravuconazole [8, 9]. Both these drugs are exceptionally potent on *T*. *cruzi in vitro* and have been shown to effect radical parasitological cure in mouse models of Chagas disease [10]. Also, treatment with posaconazole cured *T*. *cruzi* infection in an immunosuppressed patient following benznidazole treatment failure [11]. However, a 60-day treatment with posaconazole, while transiently clearing the parasite from Chagas patients, did not prevent recrudescence of infection in a majority of patients (81%) as determined by PCR. A similar trial testing the efficacy of ravuconazole prodrug E1224 has recently reported failure to cure infection in a majority (~70%) of the treated Chagas patients [12, 13]. These failures in clinical phase 2 trials have been attributed to insufficient drug exposure or dosing duration [14].

In addition to inhibitors of the parasite ergosterol biosynthesis, several inhibitors of cruzain were reported as promising candidates for treating Chagas disease. Of these, the most advanced is K777, a vinyl sulfone peptidomimetic inhibitor [15, 16]. K777 has been shown to be safe and efficacious in animal models of acute and chronic Chagas disease [17, 18] and is currently undergoing preclinical development evaluation.

Identification of *T*. *cruzi* growth inhibitors by phenotypic screening represents a viable alternative to target-based Chagas disease drug discovery. The approach allows efficient discovery of small molecules that perturb new molecular targets. Limitations of this approach stem from ignorance of the molecular mechanism, which precludes the use of structure-assisted drug design and prevents early predictions of toxicity through inhibition of homologous host enzymes. Chemical optimization of hit molecules in the absence of target-based activity measurements can be confounded by complex structure-activity-relationships, as biochemical activity and cellular permeability cannot be distinguished [19, 20].

To overcome these limitations, we have established a chemical genetics approach to the determination of the mechanism of action of small molecule growth inhibitors in *T*. *cruzi*. Starting with a novel *T*. *cruzi* inhibitor GNF7686, we evolved resistant *T*. *cruzi* mutants *in vitro*, and then identified the resistance-conferring mutation by whole genome sequencing. Finally, we demonstrated inhibition of the putative target in a biochemical assay. An expansion of this approach to other *T*. *cruzi* growth inhibitors could lead to identification of many additional drug targets and associated lead inhibitors for Chagas disease, and is already underway in our laboratory. As with the case of the *T*. *cruzi* cytochrome *b* target reported in this study, such an approach could point to drugs and drug targets from other fields, and substantially accelerate the introduction of novel Chagas disease treatments into the clinic.

## Methods

### Chemical reagents

With the exception of decylubiquinone (MP Biomedicals), all chemicals were purchased from Sigma-Aldrich Corporation.

### *T*. *cruzi* culture

*T*. *cruzi* CL strain was propagated in NIH/3T3 fibroblast cells. NIH/3T3 cells were grown in RPMI-1640 media supplemented with 10% heat-inactivated fetal bovine serum (FBS, Hyclone) and 100 IU penicillin / 100 μg streptomycin (Hyclone) per mL at 37°C / 5% CO_2_, and passaged every three to four days. To establish infection, 6.25 × 10^5^ of 3T3 cells were seeded in a T-175 flask. After attachment, cells were infected with 20–40 × 10^6^ *T*. *cruzi* trypomastigotes. Following cell infection, parasites cycled between the trypomastigote and the intracellular proliferative amastigote forms and medium was changed biweekly.

*T*. *cruzi* epimastigotes were maintained in liver infusion tryptose (LIT) medium (9 g / L liver infusion broth, 5 g / L tryptose, 1 g / L NaCl, 8 g / L Na_2_HPO_4_, 0.4 g / L KCl, and 1 g / L glucose, pH = 7.2) supplemented with 10% heat-inactivated FBS and 15 μM hemin, and passaged every five days during middle to late logarithmic growth phase (maintained at 26°C / 0% CO_2_).

### *T*. *cruzi in vitro* metacyclogenesis

For differentiation of epimastigotes into trypomastigotes, saturated cultures of *T*. *cruzi* CL epimastigotes were harvested by centrifugation (1,000 × *g* for 10 min at 10°C), resuspended in artificial triatomine urine medium (TAU; 190 mM NaCl, 17 mM KCl, 2 mM MgCl_2_, 2 mM CaCl_2_, 0.035% NaHCO_3_, 8 mM phosphate buffer, pH 6.9) at a density of 5 x 10^8^ cells / mL, and incubated at 26°C. Two hours later, the parasites were transferred to TAU 3AAG medium (TAU supplemented with 10 mM L-proline, 50 mM sodium L-glutamate, 2 mM sodium L-aspartate and 10 mM D-glucose) in T-25 culture flasks with a layer of culture medium approximately 1 cm in depth. This cell density was previously shown to be the optimal density for epimastigote differentiation [21, 22]. After 72 hour incubation, the mixture of epimastigotes and newly differentiated trypomastigotes was used for infection of mammalian host cells (NIH/3T3 mouse embryonic fibroblast line).

Briefly, NIH/3T3 cells were plated at a density of 0.025 million cells / mL into T-175 flasks and infected with 5 mL of pelleted epimastigote / trypomastigote mixture (collected from 25 mL of transformed culture) resuspended in RPMI-1640 medium supplemented with 10% FBS and 100 IU penicillin / 100 μg streptomycin per mL. After 24 hour incubation, non-internalized extracellular epimastigotes and trypomastigotes were removed, and infected NIH/3T3 cells were cultured for additional seven days. By then, newly formed trypomastigotes released from infected NIH/3T3 host cells were present in the medium and further used as described in the “*T*. *cruzi in vitro* efficacy assays” section.

### *T*. *cruzi in vitro* efficacy assays

To determine compound efficacy on *T*. *cruzi* intracellular amastigotes, NIH/3T3 cells were seeded (1,000 cells / well, 40 μL) into microscopy-grade, clear bottom, 384-well plates (Greiner) in RPMI-1640 medium containing 5% heat-inactivated fetal bovine serum and 100 IU penicillin / 100 μg streptomycin per mL. Plates were incubated overnight at 37°C / 5% CO_2_. Cells were infected with *T*. *cruzi* trypomastigotes at a multiplicity of infection (MOI) of 10 for the wild-type strain, and 20 for the GNF7686-resistant mutant strain. After six hours of infection (at 37°C / 5% CO_2_), the plates were washed by aspirating the medium and replacing with fresh screening medium to remove remaining extracellular trypomastigotes. The plates with infected cells were incubated overnight (37°C / 5% CO_2_) and compounds dissolved in DMSO were added to plate wells on the following day (0.2% DMSO final concentration). After 48-hour compound treatment, infected cells were fixed (4% paraformaldehyde in phosphate-buffered saline containing 0.5 mM CaCl_2_ and 0.5 mM MgCl_2_), permeabilized (0.1% Triton X-100 in PBS), and then stained using a 1:125,000 dilution (prepared in PBS) of SYBR green (Life Technologies). The plates were then scanned using the Evotec Opera High Content Screening System (Perkin Elmer) and amastigote proliferation was assessed by counting parasites within the 3T3 cells using CellProfiler version 2.1.0 cell image analysis software [23]. In some experiments, an alternative protocol for measurement of compound activity on intracellular *T*. *cruzi* was used [24].

To determine compound activity on the *T*. *cruzi* epimastigote form, epimastigotes (20 μL; 2.5 × 10^5^ parasites / mL) were added to 384-well assay plates containing 20 μL of LIT media with pre-dispensed compounds (0.2% DMSO final concentration) and incubated for seven days at 26°C / 0% CO_2_. Parasite viability was assessed at the end of this incubation period using the CellTiter-Glo Luminescent Cell Viability Assay (Promega). Luminescence as a measure of parasite viability was measured on the EnVision plate reader.

To assess compound efficacy on trypomastigotes, parasites (30 μL; 2 × 10^6^ trypomastigotes / mL) were added to 384-well plates containing 10 μL of RPMI-1640 without phenol red (Invitrogen) and supplemented with 10% FBS and 100 IU penicillin / 100 µg streptomycin per mL, and then treated with compounds (0.2% DMSO final concentration). Following a 48-hour incubation period, viability was assessed using the CellTiter-Glo Luminescent Cell Viability Assay (Promega).

### *Leishmania donovani in vitro* efficacy assay

*Leishmania donovani* (*L*. *donovani*) axenic amastigotes (strain MHOM/SD/62/1S-CL2D) were maintained at 37°C / 5% CO_2_ in RPMI-1640 medium containing 4 mM L-glutamine, 20% heat inactivated FBS, 100 IU penicillin / 100 μg streptomycin per mL, 23 μM folic acid, 100 M adenosine, 22 mM D-glucose, and 25 mM 2-(N-morpholino)ethanesulfonic acid (pH 5.5 adjusted at 37°C using 1M HCl).

For compound screening, axenic amastigotes were seeded into 384-well plates containing axenic amastigote medium with pre-dispensed compounds (0.25% final DMSO concentration) at a density of 9,600 cells / well. Plates were incubated for 48 hours at 37°C / 5% CO_2_. Cell viability was assessed using the CellTiter-Glo Luminescent Cell Viability Assay (Promega).

### *Trypanosoma brucei in vitro* efficacy assay

*Trypanosoma brucei brucei* (*T*. *b*. *brucei*) bloodstream form (strain Lister 427) was maintained in HMI-9 medium: IMDM (Iscove's Modified Dulbecco's Media), 10% heat-inactivated FBS, 10% Serum Plus medium supplement (SAFC biosciences), 1 mM hypoxanthine, 50 μM bathocuproine disulfonate.Na_2_, 1.5 mM cysteine, 1 mM pyruvate, 39 μg/mL thymidine, and 0.2 mM 2-mercapthoethanol) at 37°C / 5% CO_2_.

For compound screening, parasites were seeded into 384-well assay plates containing HMI-9 with pre-dispensed compounds (0.25% final DMSO concentration) at a density of 6,000 cells / well, and plates were then incubated for 48 hours at 37°C / 5% CO_2_. Cell viability was assessed using the CellTiter-Glo Luminescent Cell Viability Assay (Promega).

For conversion from the bloodstream form to the procyclic form, bloodstream form parasites were transferred from HMI-9 medium to Differentiating Trypanosome Medium (DTM, pH = 7.2), consisting of: 6.8 g / L NaCl, 400 mg / L KCl, 200 mg / L CaCl_2_, 140 mg / L NaH_2_PO_4_.H_2_O, 200 mg / L MgSO_4_.7H_2_O, 7.94 g / L HEPES, 2.2 g / L NaHCO_3_, 110 mg / L sodium pyruvate, 10 mg / L phenol red, 14 mg / L hypoxanthine, 1 mg / L biotin, 760 mg / L glycerol, 640 mg / L proline, 236 mg / L glutamic acid, 1.34 g / L glutamine, 7.5 mg / L hemin (in 50 mM sodium hydroxide), 1X MEM amino acid solution (Invitrogen),1X MEM non-essential amino acids solution (Invitrogen), 28.2 mg / L bathocuproine disulfonate.Na_2_, 182 mg/L cysteine, 0.2 mM 2-mercaptoethanol, 15% heat-inactivated FBS, and 5 mM sodium citrate and cis-aconitate [21, 22]. Following medium exchange, parasites were incubated at a lower temperature (27°C / 5% CO_2_), monitored for change in morphology to procyclic parasites, and sub-cultured for long-term cultivation.

For compound screening, 20 μliters (5,000 parasites) of *T*. *b*. *brucei* procyclic culture were added to 384-well plate wells filled with 20 μliters of DTM medium and pre-dispensed compounds (0.25% final DMSO concentration). Plates were then incubated for 72 hours at 27°C / 5% CO_2_. Cell viability was assessed using the CellTiter-Glo Luminescent Cell Viability Assay (Promega).

### *Plasmodium falciparum in vitro* efficacy assay

GNF7686 and cytochrome *b* inhibitors were assayed for activity on two *Plasmodium falciparum* (*P*. *falciparum*) lines: D10attB and yDHODH-D10attB. The D10attB line is reliant on the coenzyme Q-dependent malarial dihydroorotate dehydrogenase (*Pf*DHODH), whereas the yDHODH-D10attB line expresses also the fumarate-utilizing *Saccharomyces cerevisiae* (*S*. *cerevisiae*) DHODH, which circumvents reliance on *Pf*DHODH and renders this line fully resistant to cytochrome *b* inhibitors [25]. The use of these two lines allows for distinction of selection of potent cytochrome *b* inhibitors as described in detail in the Results section [26, 27]. Growth and viability of *P*. *falciparum* cell lines (in the presence or absence of drug) in infected erythrocytes were assessed using a SYBR Green-based proliferation assay exactly as described previously [28].

### Mammalian cell cytotoxicity assay

NIH/3T3 fibroblast cells were maintained in RPMI-1640 medium supplemented with 10% heat-inactivated FBS and 100 IU penicillin / 100 μg streptomycin per mL at 37°C / 5% CO_2_. For compound screening, cells were diluted to 4 × 10^4^ cells / mL in assay medium (RPMI-1640, 5% FBS, and 100 IU penicillin / 100 μg streptomycin per mL) and seeded at 50 μL / well into 384-well plates. Following overnight incubation, compounds were added to each well (0.2% DMSO final concentration) and plates were further incubated for 96 hours. Cell viability was assessed using the CellTiter-Glo Luminescent Cell Viability Assay (Promega). Measured luminescence values were normalized to the value obtained for 0.2% DMSO, and plotted against the corresponding compound concentration for half maximal cytotoxic concentration (CC_50_) value determination.

### *S*. *cerevisiae* susceptibility assay

The minimal inhibitory concentrations (MIC) of the compounds for inhibition of *S*. *cerevisiae* drug pump knock-out strain NF7061 (*MATa his3Δ 1; leu2Δ 0; met15Δ 0; ura3Δ 0; snq2*::*KanMX; pdr5*::*KanMX; pdr1*::*NAT1; pdr3*::*KanMX; yap1*::*NAT1; pdr2*::*LEU2; yrm1*::*MET; yor1*::*LYS2*) were determined by a modification of the microdilution technique described elsewhere [29]. Briefly, two-fold dilutions of the compound solution in DMSO were made. They were added to each well of 96-well assay plate containing 200 μL per well of either YPD (1% Difco Yeast Extract, 2% Difco Bacto Peptone and 2% Dextrose) or YPG (3% of glycerol in replacement of 2% Dextrose in YP) medium. Early stationary yeast NF7061 cells grown in either medium were collected and resuspended to 2 × 10^5^ cells / mL. Ten microliters of the yeast cell suspension were inoculated to each well of the plates containing medium and compound to achieve a final inoculum of approximately 10^4^ CFU / mL. The plates were incubated at 30°C for 24 hours (containing YPD) or 48 hours (containing YPG). The MIC end point was defined as the lowest compound concentration exhibiting no visual growth.

### Selection of GNF7686-resistant *T*. *cruzi* and cloning

*T*. *cruzi* epimastigotes were initially treated with GNF7686 at EC_20_ value (0.01 μM, 0.2% DMSO) and continually passaged at the same concentration until the culture growth rate matched that of epimastigotes growing in the medium containing 0.2% DMSO. Parasites were subsequently passaged in a similar manner in the presence of increasing concentration of GNF7686 until significant resistance was achieved (~5-fold increase in the EC_50_ value). The time to generate resistance was approximately eleven months. Resistant epimastigotes were cloned by the limiting dilution technique.

### Whole genome sequencing

Following expansion of GNF7686-resistant *T*. *cruzi* clones in LIT media, *T*. *cruzi* total DNA was isolated using Qiagen DNeasy Blood and Tissue Kit from 10^8^ parasites per sample. Whole genome sequencing was performed using Ilumina HiSeq1000 next-generation sequencing platform.

Sequencing reads were aligned by Burrows-Wheeler Aligner (BWA, version 5.9.0) to the *T*. *cruzi* JR cl. 4 genome (version 1.0). Simple single nucleotide variants (SNVs) were called (using Samtools 1.19) looking for SNVs or small indels with an overall quality > 100 where the control was the drug-sensitive parental CL clone. Approximately 600900 reads called a ‘T’ and 520 reads called a “G” at L197F position resulting in L197F mutation. Heterozygous calls were determined by Samtools, and verified in the Integrated Genomics Viewer. Putative SNVs were manually checked in IGV [30, 31].

To further confirm the presence of L197F mutation, the *T*. *cruzi* cytochrome *b* gene was amplified by PCR (forward primer 5’-AGCTACTGTTCCTGTATTCGGC-3’ and reverse primer 5’-ACAAAAACAAAGTCGCTCACAA-3’) and cloned into the pCR2.1 vector. The insert DNA was sequenced using M13R and M13F (-21) primers (Genewiz).

### Measurement of *T*. *cruzi* epimastigote respiration

GNF7686 and cytochrome *b* inhibitors (0.2% DMS0 final concentration) were added to 384-well assay plates containing 20 μL of assay buffer (250 mM sucrose, 15 mM KCl, 5 mM MgCl_2_, 1 mM EGTA, 30 mM K_2_HPO_4_, pH 7.4) and allowed to dissolve for two hours. Meanwhile, *T*. *cruzi* epimastigotes were harvested (800 × g for 5 min at 4°C), washed twice with buffer A (10 mM Tris-HCl, pH = 7.4, 0.23 M mannitol, 0.07 M sucrose, 0.2 mM EDTA, 0.2% bovine serum albumin, 0.5 mM phenylmethanesulfonyl fluoride), and finally resuspended in buffer A at a final concentration of 150 × 10^6^ epimastigotes / mL. Next, 20 μL of *T*. *cruzi* epimastigote suspension (3 x 10^6^ parasites) was added to each sample well, and then 15 μL of MitoXpress-Xtra probe (Cayman Chemicals) was added (final assay volume of 60 μL). Probe phosphorescence is quenched in the presence of oxygen and is inversely proportional to the amount of oxygen present in the solution. HS mineral oil (20 μL, Cayman Chemicals) was added to wells to prevent oxygen exchange between the assay buffer and air. Blank wells containing assay buffer without parasites, corresponding compound, and mineral oil were prepared in parallel and values were subtracted from sample values to specifically measure changes in oxygen concentration caused by parasite respiration. All sample and blank wells were prepared in duplicate. The assay plate was transferred to a Gemini XPS fluorescence plate reader (Molecular Devices) and read at 3 minute intervals at excitation and emission wavelengths of 380 nm and 650 nm, respectively, for 30 minutes at 37°C. The slope for the linear portion of time course of fluorescence increase (rate of oxygen consumption) was calculated after subtraction of blank well values. Obtained slope values were normalized to the slope obtained for 0.2% DMSO, and plotted against the corresponding compound concentration for half maximal inhibitory concentration (IC_50_) value determination.

### Measurement of *T*. *cruzi* complex III activity

*T*. *cruzi* epimastigotes (57 day old culture, density of 5 × 10^7^ parasites / mL) were harvested by centrifugation (800 × g for 5 minutes at 4°C) and resuspended at 10 mg / mL of protein in buffer A containing 0.1 mg digitonin / mg protein. The parasites were then incubated with the detergent for 10 minutes at 26°C. The pellet fraction was collected by centrifugation (13,000 × g for 5 min at 4°C) and immediately used in the complex III assay.

Complex III activity was monitored using a coupled decylubiquinol / cytochrome *c* reaction [32, 33]. Decylubiquinone was first reduced to decylubiquinol as described [33]. The freshly reduced decylubiquinol (80 μM final concentration in the reaction) was added to a reaction buffer (25 mM potassium phosphate, 5 mM MgCl_2_, 2.5 mg / mL BSA, pH = 7.2) containing 1 mM KCN, 0.1 mM yeast cytochrome *c*, 0.6 mM n-D-B-maltoside, and 12 μg of digitonin-solubilized *T*. *cruzi* epimastigotes. The reduction of cytochrome *c* was monitored at 550 nm using the SpectraMax Plus384 absorbance microplate reader. Blank samples containing all components excluding decylubiquinol were processed in parallel and absorbance values were subtracted from sample absorbance values to specifically measure decylubiquinone-dependent reduction of cytochrome *c*. All sample and blank wells were prepared in triplicate. For IC_50_ determination, the slope of the linear portion of the corrected respiration trace was determined, and normalized to the slope obtained for 0.2% DMSO condition.

### Preparation of rat skeletal muscle mitochondria

Sprague Dawley rats were euthanized and skeletal muscle (from hind legs) was removed and stored in CP1 buffer (0.1 M KCl, 0.05 M Tris, 2 mM EGTA, pH 7.4 at 4°C) on ice. Using the ‘Herb Mincer‘, tissue was minced 34 times and then transferred into CP1 buffer (on ice) to wash away fatty and connective tissues from muscle. Following two rounds of wash and decantation with CP1 buffer, the rinsed tissue was transferred to CP2 buffer (CP1 + 0.5% BSA, 5 mM MgCl_2_, 1 mM ATP, 250 units / 100 mL Protease Type VIII (Sigma P5380), pH 7.4 at 4°C) and incubated on ice for 5 minutes prior to homogenization using the Polytron PT3100. Following homogenization and centrifugation (500 × g for 10 min at 4°C), the supernatant was decanted using cheesecloth and further centrifuged (10,000 × g for 11 min at 4°C). The crude mitochondrial pellet was resuspended in 10 mL of CP1 buffer (carefully avoiding red blood cell pellet) and subjected to an additional centrifugation step (10,000 × g for 10 min at 4°C). The resulting pellet was again separated from the red blood cell pellet and centrifuged (600 x g for 6 min at 4°C). The supernatant containing resuspended mitochondria was subjected to one final round of centrifugation (5,000 × g for 11 min at 4°C) and the pelleted mitochondria were resuspended in CP1 buffer and stored at ^-^80°C. Respiration in isolated rat mitochondria was measured using the same protocol as described for *T*. *cruzi* epimastigotes using 150 μg protein / sample.

## Results

### Characterization of anti-parasitic activity of GNF7686 HTS hit

**Fig 1:**
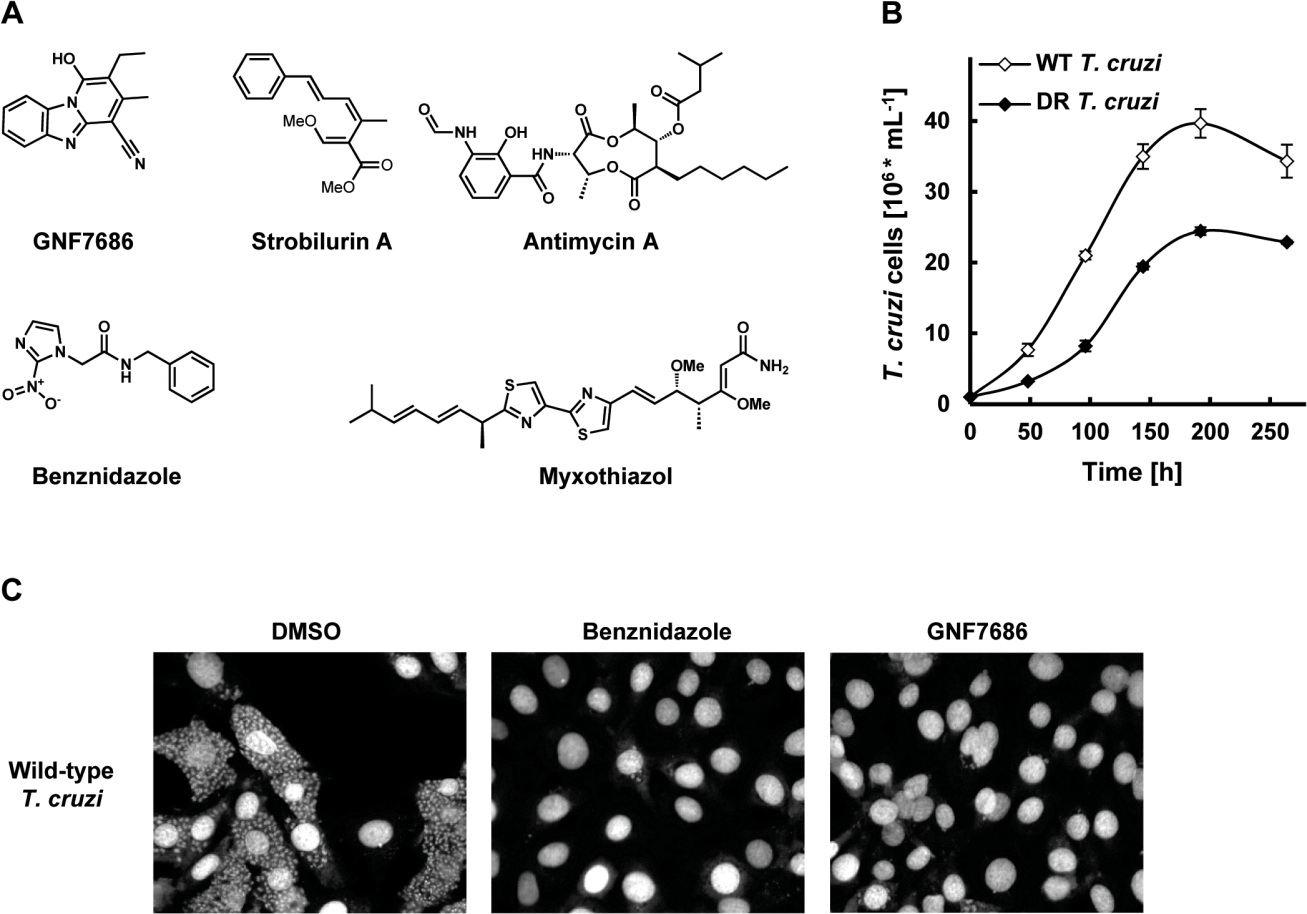
GNF7686 clears *T*. *cruzi* amastigotes from infected 3T3 cells. A) Structures of GNF7686, benznidazole and prototypic cytochrome *b* inhibitors used in this study. B) Growth curves of wild-type and GNF7686-resistant *T*. *cruzi* epimastigotes. C) Microscopy images of NIH/3T3 cells infected with *T*. *cruzi* after 48 hour treatment with 0.2% DMSO, 5 μM benznidazole or 1 μM GNF7686.

GNF7686 (Fig 1A) was identified in a high throughput screen designed to find new small molecules with growth inhibition activity on *L*. *donovani* axenic amastigotes. A library of 700,000 compounds was assembled with a particular focus on drug-like properties and structural diversity and these compounds were tested for inhibitory activity on *L*. *donovani* at 4 μM concentration. The library has been previously profiled in more than 60 other high throughput screens, including biochemical and cell-based assays against human and pathogen targets. The screen history allowed rapid identification and elimination of compounds with a ‘frequent hitter’ property.

The screen yielded 2,306 primary hits (0.3% hit rate) that inhibited growth by > 50%. Data from more than 95% of the assay plates had Z′ > 0.7, using DMSO as the negative control and 5 μM pentamidine as the positive control. Primary hits from the screen were further characterized using a dose−response assay format to determine the EC_50_ values. In parallel, cytotoxicity of these compounds was determined against a proliferating mouse fibroblast cell line (NIH/3T3). The final set of condirmed hits consisted of compounds that had EC_50_ < 4 μM against *L*. *donovani*, as well as low or no 3T3 cytotoxicity (CC_50_ > 10 μM or SI > 10; SI = CC_50_/EC_50_). The final set of confirmed *L*. *donovani* hits consisted of 1003 inhibitors.

The confirmed hits were further assayed for activity on other two medically important kinetoplastid parasites *T*. *cruzi* and *T*. *brucei*. GNF7686 was selected for further investigation because of potent *in vitro* activity on all three *T*. *cruzi* morphological forms (intracellular amastigote EC_50_ = 0.15 μM, trypomastigote EC_50_ = 0.71 μM, epimastigote EC_50_ = 0.16 μM; Table 1 and Fig 1C). GNF7686 also inhibited the growth of *L*. *donovani* axenic amastigotes (EC_50_ = 0.46 μM) and promastigotes (EC_50_ = 0.46 μM), but not the growth of *T*. *b*. *brucei* bloodstream form trypomastigotes (EC_50_ > 25 μM). Curiously, GNF7686 was active on *T*. *b*. *brucei* procyclics (EC_50_ = 0.59 μM), the parasite form found in the tsetse fly vector that mediates *T*. *b*. *brucei* transmission [34]. GNF7686 did not inhibit growth of 3T3 cell line (CC_50_ > 20 μM).

**Table 1:** Potency of GNF7686 and prototypic cytochrome *b* Inhibitors on wild-type (WT) and GNF7686-resistant (DR) *T*. *cruzi* morphological forms.

	Amastigote EC_50_ [μM]		Epimastigote EC_50_ [μM]		Trypomastigote EC_50_ [μM]	
Compound	WT	DR	WT	DR	WT	DR
GNF7686	0.15 ± .03*	0.66 ± 0.12*	0.16 ± 0.02*	0.73 ± 0.04*	0.71 ± 0.16*	4.5 ± 0.84*
Antimycin A	N.A.	N.A.	0.046 ± 0.01*	1.8 ± 0.21*	0.20 ± 0.04*	4.3 ± 1.3*
Myxothiazol	N.A.	N.A.	0.49 ± 0.05	0.43 ± 0.08	2.9 ± 0.48	2.9 ± 0.43
Strobilurin	N.A,	N.A.	0.59 ± 0.13	0.34 ± 0.01	1.7± 0.41	1.4 ± 0.36
Benznidazole	1.4 ± 0.17	0.82 ± 0.13	5.5 ± 0.15	6.7 ± 0.6	13± 1.8	11 ± 0.58

EC_50_ values were calculated from three independent repeats (n = 3), each performed in duplicate. Standard error values are also shown. N.A. in the table stands for ‘not applicable’.

*P-value for wild-type versus drug-resistant *T*. *cruzi* EC_50_ values is < 0.05.

### Identification and characterization of GNF7686-resistant *T*. *cruzi*

To investigate the mechanism of action of GNF7686, we selected a population of drug-resistant *T*. *cruzi* epimastigotes through a long-term parasite culture in the presence of this compound. As tolerance for GNF7686 gradually increased over time, we periodically escalated the selection pressure by raising the inhibitor concentration. In the course of eleven months, the EC_50_ of the *T*. *cruzi* culture shifted ~ 4-fold from 0.16 µM to 0.73 μM (Table 1).

Populations of evolving microbes often comprise cells harboring alternative mutations that are derived from different mutation events [35, 36]. To simplify analysis of genomic changes accumulated during the selection by characterizing homogenous culture populations, we isolated three clones from the GNF7686-resistant culture. All three clones exhibited the same extent of GNF7686 resistance (EC_50_ ~ 1 μM) as the parent culture. Importantly, the sensitivity of clones to benznidazole remained at the same level as observed for the wild-type *T*. *cruzi* strain (Table 1), demonstrating that resistance to GNF7686 did not arise through a broadly pleiotropic mechanism. When cultured in medium lacking GNF7686, all three clones grew at a rate similar to the wild-type strain (mutant epimastigote doubling time = 5355 hours vs 60 hours for the wild-type strain), but reached stationary phase at a lower cell density (~60% of the wild-type strain, Fig 1B).

Whole genome sequencing identified the same set of five mutations in all three clones that included L197F in cytochrome *b*, L283M in the ATPase subunit of HsIVU protease, R75C in TCSYLVIO008926 hypothetical protein, and two mutations in non-coding regions. The observation that the three clones were identical at the genome sequence level suggests that they were siblings derived from one founding cell that expanded in the passaged culture during the selection. During whole genome sequencing, multiple sequence reads (up to 100 in total) from many independent DNA molecules are obtained for each nucleotide position in the genome. We uncovered that the L197F mutation in the cytochrome *b* gene and one of two mutations mapped to non-coding regions fully replaced the corresponding wild-type alleles, while the other three mutations remained heterozygous during the selection. Interestingly, the two mutations map both to the kinetoplast maxicircle DNA, which is present in 2050 copies per cell [37, 38]. This indicates that these two mutations, presumably appearing on one maxicircle copy at first, replaced the corresponding wild-type alleles during the selection to the point of achieving homoplasmy.

### Chemogenomic profiling of GNF7686 in yeast supports inhibition of the respiratory chain at the level of complex III

In addition to selection *of T*. *cruzi* mutants resistant to GNF7686, we subjected the inhibitor also to chemogenomic profiling in *S*. *cerevisiae*. The genome-wide deletion collections available for this eukaryotic model system provide powerful genetic tools for investigation of bioactive molecules [39, 40], and the approach was successfully applied to mechanism of action studies of various growth inhibitors in the past [41, 42]. In the haploinsufficiency profiling assay (HIP), complete collection of heterozygous yeast deletion strains, in which each strain has only one copy of a particular gene, is profiled for hypersensitivity to a compound. Gene deletions associated with increased compound sensitivity indicate pathways directly affected by the compound [43].

**Fig 2:**
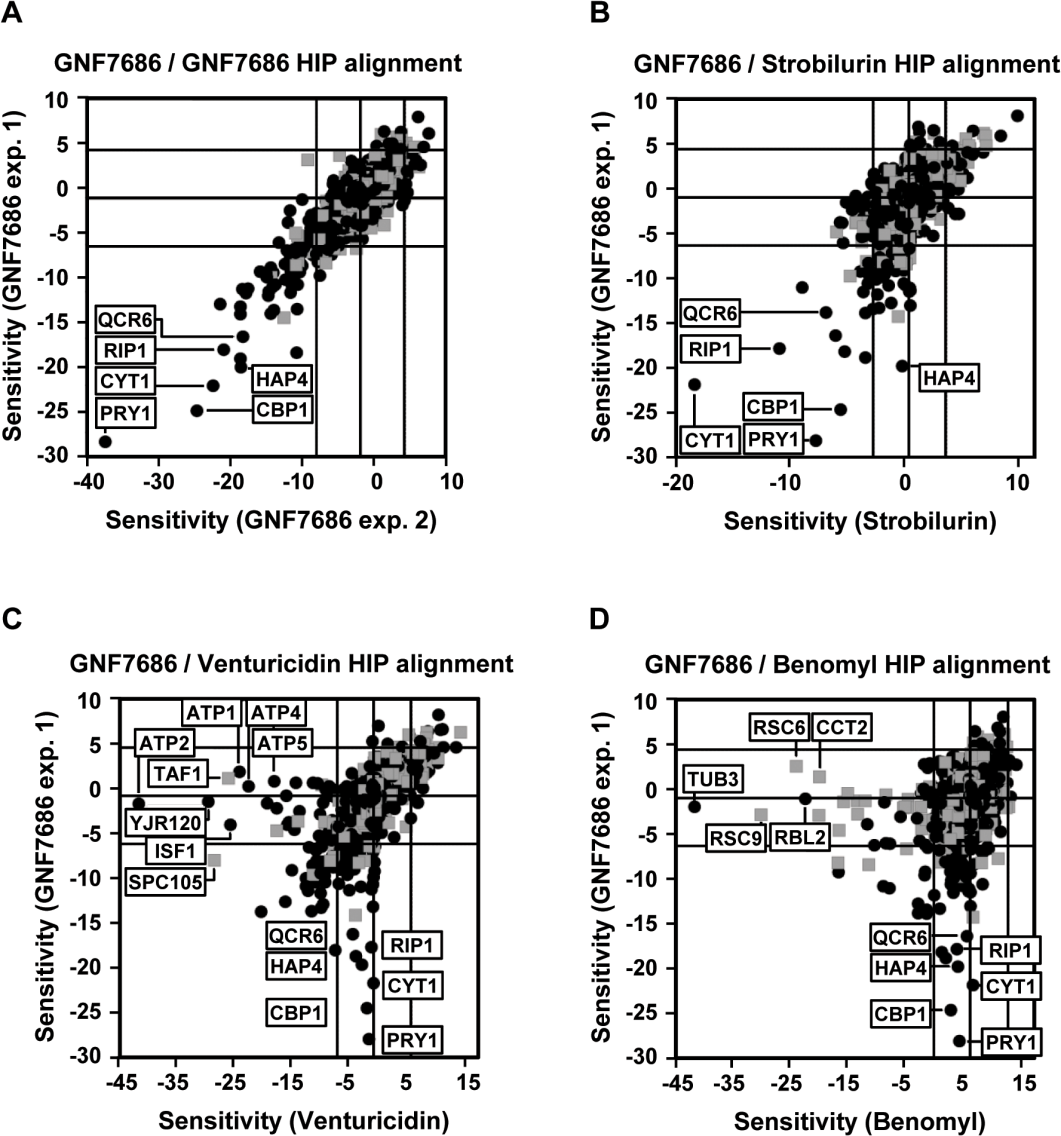
Chemogenomic profiling in *S*. *cerevisiae* suggests cytochrome *b* as the target of GNF7686 in yeast. A) HIP profile of GNF7686. B-D) Alignment of the HIP profile of GNF7686 with profile of cytochrome *b* inhibitor strobilurin (B), F-type ATPase inhibitor venturicidin (C), and microtubule-binding fungicide benomyl (D).

We observed that growth of *S*. *cerevisiae* is inhibited when the yeast was cultured in media containing glycerol but not glucose as the carbon source (see also below). We therefore conducted a HIP profiling experiment in medium containing glycerol. Testing of GNF7686 at its EC_30_ concentration in two independent HIP experiments resulted in a reproducible profile (Fig 2A). Identified hypersensitive heterozygous strains included those with deletions in genes involved in mitochondrial metabolism, such as *CYT1* (cytochrome *c*_*1*_), *HAP4* (a transcription factor involved in regulation of the respiratory chain including *CYT1*), *CBP1* (a regulator of cytochrome *b* mRNA stability), and *QCR6* (a subunit of the cytochrome *c* reductase complex) [44]. As all these hits pointed at inhibition of mitochondrial respiration by GNF7686, we performed additional HIP experiments with strobilurin, an inhibitor of cytochrome *bc*_*1*_ complex, and venturicidin, an inhibitor of F-type ATPase [45, 46]. In a control experiment, we also collected the HIP profile for benomyl, a microtubule binding inhibitor, which does not interfere with ATP production during oxidative phosphorylation.

While both venturicidin and benomyl yielded HIP profiles distinctly different from that of GNF7686 (Fig 2C and 2D), treatment of gene deletion strain collection with the cytochrome *bc*_*1*_ inhibitor strobilurin identified essentially the same set of sensitive, heterozygous mutants as GNF7686 and included *CYT1*, *HAP4*, *CBP1* and *QCR6* (Fig 2B). It is important to note that the gene coding for cytochrome *b*, which is the proposed direct target of strobilurin [47], is encoded by the mitochondrial genome in *S*. *cerevisiae* and not amenable to standard gene targeting protocols. Thus, the cytochrome *b* gene deletion strain is not present in the yeast heterozygous deletion pool and could not be directly identified by the HIP method.

The observed HIP compound profiles strongly suggested that GNF7686 directly interferes with function of the *S*. *cerevisiae* respiratory chain, possibly at the level of complex III.

### Prototypical cytochrome *b* inhibitors block *in vitro* proliferation of *T*. *cruzi*

**Fig 3:**
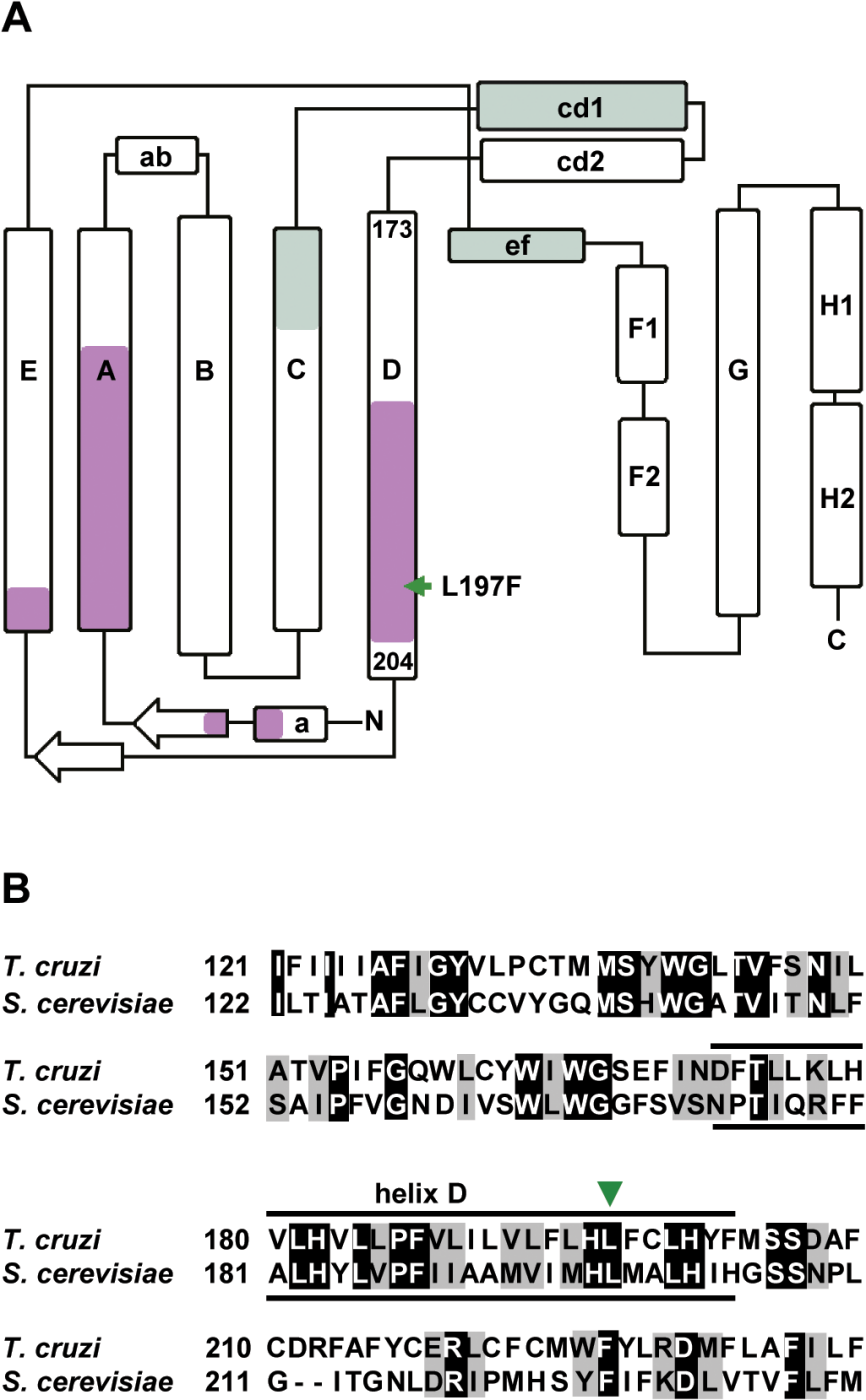
GNF7686 resistance-conferring mutation in *T*. *cruzi* cytochrome *b* structure. A) Secondary structure of yeast cytochrome *b* (adapted from Ding *et al*. 2006 and Fisher *et al*. 2008, [50, 57]) with amino acid sequence stretches that form Q_P_ and Q_N_ ubiquinol-binding sites highlighted (green and violet, respectively). The amino acid indicated by a green arrow corresponds to the L197F mutation (equivalent to L198F in *S*. *cerevisiae*) present in the GNF7686-resistant *T*. *cruzi* strain. B) Alignment of *S cerevisiae* and *T*. *cruzi* cytochrome *b* amino acid sequence around L197F mutation. Identical amino acids are highlighted in black and conserved substitutions are highlighted in grey.

Genomic analyses of GNF7686 resistance/sensitivity pointed to involvement of the *T*. *cruzi* cytochrome *b* in resistance to growth inhibition by GNF7686. Cytochrome *b* is a component of the multisubunit cytochrome *bc*_1_ complex, an asymmetric homodimer with two spatially separated catalytic sites Q_N_ and Q_P_ (Fig 3A). In concert, Q_N_ and Q_P_ catalyze oxidation of ubiquinol formed by preceding steps of the respiratory chain [48, 49].

Inhibitors of cytochrome *b* are already of interest as anti-parasitic drugs. Atovaquone, a hydroxy-naphthoquinone inhibitor of the Q_P_ site, is used in the treatment of malaria and fungal pneumonia [50]. Another hydroxy-naphthoquinone, buparvaquone, is used to treat cattle theileriosis, and potently inhibits growth of *L*. *donovani* [19, 51]. Surprisingly, the potential of cytochrome *b* inhibitors for treatment of Chagas disease has not been explored, even though cytochrome *b* inhibitors including antimycin A were shown to affect *T*. *cruzi* mitochondrial respiration, bioenergetics, and calcium homeostasis [52–55]. To assess the effect of this class of compounds on *T*. *cruzi* growth and survival, we tested prototypical Q_N_ and Q_P_ site inhibitors on intracellular amastigotes, trypomastigotes, and epimastigotes (inhibitor structures shown in Fig 1A) [46, 49].

The Q_N_ site inhibitor antimycin A potently inhibited the growth of epimastigotes and rapidly reduced viability of trypomastigotes. We also observed *T*. *cruzi* inhibition by compounds targeting the cytochrome *b* Q_P_ site. Two well-characterized Q_P_ site inhibitors, myxothiazol and strobilurin, blocked growth of epimastigotes, and reduced viability of trypomastigotes (Table 1). The effect of antimycin A, myxothiazol, and strobilurin on intracellular amastigotes could not be accurately determined because of the inhibitor toxicity on the host 3T3 cells.

*T*. *b*. *brucei* is a kinetoplastid parasite closely related to *T*. *cruzi* at the genomic level [56]. While the parasite bloodstream (mammalian) form of *T*. *b*. *brucei* relies primarily on the glycolytic pathway for ATP production, growth of the procyclic (insect) form requires activity of the conventional respiratory pathway, including cytochrome *b* [48]. In line with the hypothesis that GNF7686 is a cytochrome *b* inhibitor, GNF7686 inhibited the growth of the procyclic but not bloodstream *T*. *b*. *brucei* parasites (EC_50_ = 0.59 μM vs > 25 μM). We also observed similar differential activity on the two *T*. *brucei* forms with the other tested cytochrome *b* inhibitors such as antimycin A (EC_50_ = 0.03 μM vs > 25 μM; Table 2).

**Table 2:** Potency of GNF7686 and prototypic cytochrome *b* inhibitors in *L*. *donovani*, *T*. *brucei*, *P*. *falciparum* and *S*. *cerevisiae* proliferation assays.

	*L*. *donovani* EC_50_ [μM]		*T*. *b*. *Brucei* EC_50_ [μM]		*P*. *falciparum* EC_50_ [μM]		*S*. *cerevisiae* EC_50_ [μM]	
Compound	Amastigote	Promastigote	Procyclic	BSF	D10attB	yDHODH-D10attB	YPD medium	YPG medium
GNF7686	0.46 ± 0.28	0.46 ± 0.12	0.59 ± 0.09	>25	>12.5	>12.5	>40	5.0±0.0
Antimycin A	0.01 ± 0.004	0.01 ± 0.003	0.03 ± 0.002	>25	0.70±0.34	>12.5	5.0±0.0	< 0.002
Myxothiazol	9.6 ± 3.9	3.1 ± 0.45	2.5 ± 0.22	17.2 ± 3.1	0.3±0.01	8.4±1.6	30±10	0.07±0.01
Strobilurin	9.1 ± 2.5	4.3 ± 0.82	3.2 ± 0.33	>25	0.46±0.27	>12.5	>40	0.1±0.02
Benznidazole	>25	>25	>25	>25	>12.5	>12.5	>40	>40

GNF7686 and cytochrome *b* inhibitors were tested for cytotoxicity in various parasites using the CellTiter-Glo Luminescent Cell Viability assay reagent system (see Table 1), SYBR Green Fluorescent dye (*P*. *falciparum* only), or MIC visual growth inhibition screen (*S*. *cerevisiae* only). Luminescence or fluorescence was monitored using the EnVision Multilabel Plate reader and EC_50_ values (with respective standard errors) were determined based on three (n = 3; *L*. *donovani* and *T*. *b*. *brucei*) or two (n = 2; *P*. *falciparum* and *S*. *cerevisiae*) biological repeats with duplicate technical repeats. Abbreviations include: BSF (bloodstream form), DHODH (malarial dihydroorotate dehydrogenase), yDHODH-D10attB (fumarate-utilizing *S*. *cerevisiae* DHODH), YPD (yeast extract peptone with dextrose), and YPG (yeast extract peptone with glycerol).

The inhibition of various morphological forms of *T*. *cruzi* by prototypical cytochrome *b* inhibitors is consistent with the hypothesis that the cytochrome *b* fulfills an essential function in parasite physiology and that GNF7686 inhibits its function. However, with the exception of antimycin A, the anti-parasitic potency of the other tested inhibitors is too weak to be of therapeutic significance.

### GNF7686 is an inhibitor of cytochrome *b* Q_N_ site

Through a literature search, we identified a previously published report that described L198F mutation in *S*. *cerevisiae* cytochrome *b*, which is equivalent to the L197F mutation in *T*. *cruzi* cytochrome *b* (Fig 3B). The yeast L198F mutation confers resistance to ilicicolin H, a cytochrome *b* inhibitor with potent anti-fungal activity [46, 57]. Inspection of high resolution crystal structure of the yeast cytochrome *bc*_*1*_ complex further revealed that the Leu198 side chain is positioned in close proximity (< 5 Å) to ubiquinol bound inside the Q_N_ pocket and next to His197, which coordinates the iron atom in the b_H_ heme. In accordance with the structure, L198F mutation also conferred resistance in yeast to other tested Q_N_ site inhibitors such as funiculosin and antimycin A [57, 58].

Additional characterization of the GNF7686-resistant *T*. *cruzi* mutants revealed that they were selectively resistant to antimycin A, a Q_N_ site inhibitor [46]. While antimycin A displayed very potent activity on wild type epimastigotes, a sharp decrease in potency (40-fold) was observed with GNF7686-resistant epimastigotes (EC_50_ = 1.8 μM, Table 1). Similarly, a steep shift in potency (20-fold) was observed between the wild-type and mutant trypomastigotes (Table 1). In contrast, Q_P_ inhibitors myxothiazol and strobilurin showed comparable activity on both wild-type and resistant strains (Table 1). In summary, the L197F mutation in the *T*. *cruzi* cytochrome *b* is likely located within the Q_N_ site and can interfere with binding of Q_N_ site inhibitors in a similar way as was previously described for the L198F mutation in the *S*. *cerevisiae* cytochrome *b*.

### GNF7686 inhibits cytochrome *b* and respiration of *T*. *cruzi*

During aerobic respiration, the electron transport chain (ETC) conducts electrons derived from reduced carbon substrates through a series of redox reactions to the terminal electron acceptor, molecular oxygen, which is then reduced to water [33, 48]. Inhibition of electron flow through the ETC at any step, including cytochrome *b*, results in a block of oxygen consumption.

**Fig 4:**
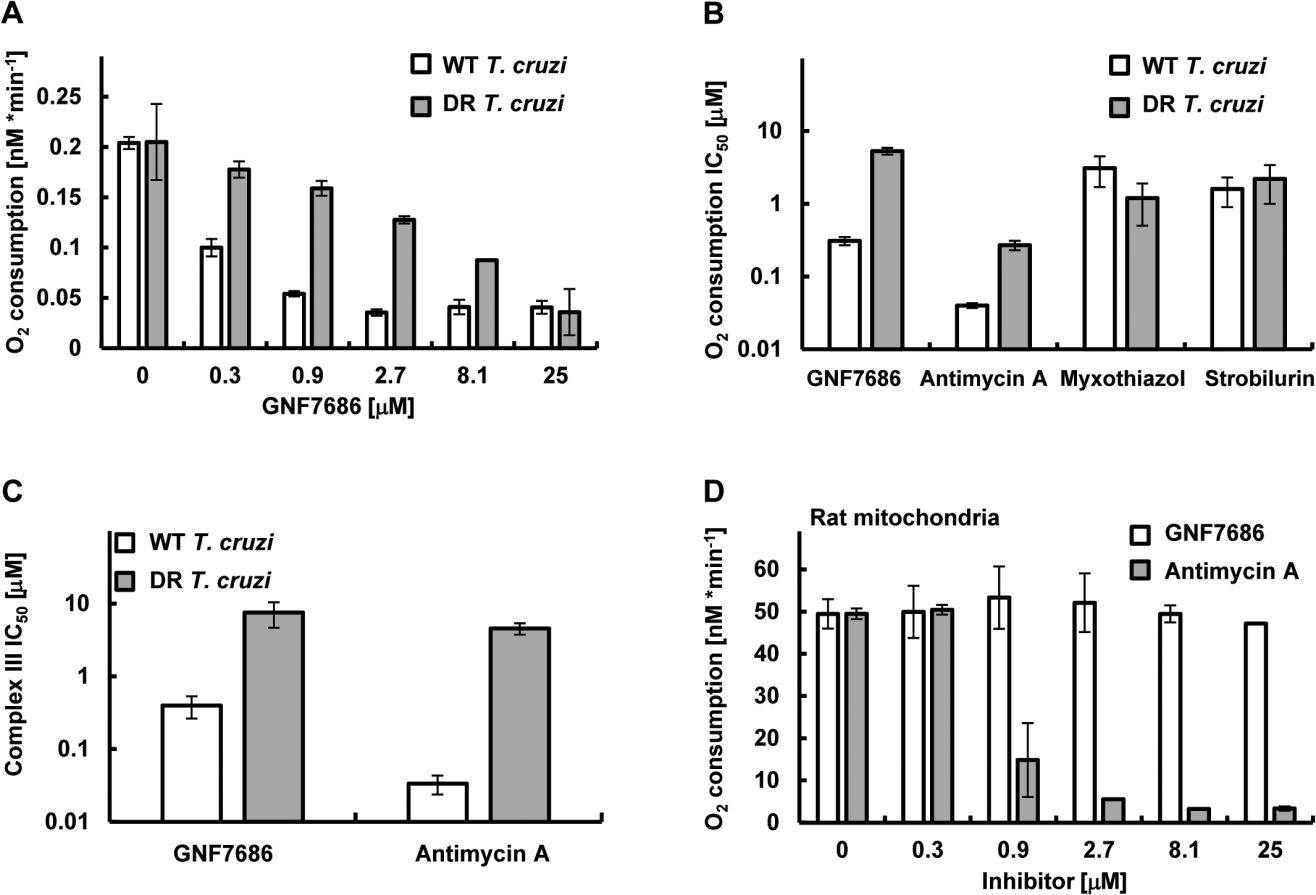
GNF7686 inhibits cellular respiration and cytochrome *b* function in *T*. *cruzi*. A) *T*. *cruzi* epimastigote respiration was monitored using the MitoXpress-Xtra HS phosphorescent probe (see Methods) in the presence of 0.2% DMSO (control) and various concentrations of GNF7686. Oxygen consumption rates per 10^6^ *T*. *cruzi* epimastigotes cells are shown. Plotted values were derived from three biological repeats (n = 3) with duplicate technical repeats in either wild-type or evolved GNF7686-resistant (DR) parasites. B) Oxygen consumption IC_50_ values for prototypic cytochrome *b* inhibitors *in T*. *cruzi* epimastigote respiration assay on wild-type and GNF7686-resistant (DR) parasites. Shown GNF7686 IC_50_ values were derived from the experiment shown in the (A) panel. C) Mitochondrial complex III activity was monitored in digitonin-solubilized *T*. *cruzi* epimastigotes (both wild-type and evolved GNF7686-resistant (DR) parasite strains) utilizing a coupled decylubiquinol oxidation / cytochrome *c* reduction reaction in the presence of a compound (GNF7686 or antimycin A). IC_50_ values were determined based on three biological repeats (n = 3) with triplicate technical repeats in either wild-type or evolved drug-resistant (DR) parasites relative to 0.2% DMSO conditions. D) High selectivity of GNF7686 for *T*. *cruzi* cytochrome *b* is reflected in the absence of inhibition of rat mitochondrial respiration by this compound up to 25 μM concentration. For comparison, antimycin A potently inhibits mammalian mitochondrial respiration. Oxygen consumption rates per 1 mg of total mitochondrial protein are shown.

To evaluate whether GNF7686 disrupts the function of the *T*. *cruzi* ETC, oxygen consumption by intact *T*. *cruzi* epimastigotes was monitored in the presence of GNF7686 and prototypic cytochrome *b* inhibitors (Fig 4A and 4B). Antimycin A potently blocked respiration by wild-type epimastigotes (IC_50_ = 0.04 μM), but was ~7-fold less potent on GNF7686-resistant parasites (IC_50_ = 0.27 μM). Two Q_P_ site inhibitors employed in this report, myxothiazol and strobilurin, both inhibited epimastigote respiration, but, in contrast to antimycin A, the respiratory IC_50_ values of the Q_P_ inhibitors were comparable between wild-type and GNF7686-resistant *T*. *cruzi*.

GNF7686 inhibited respiration by wild-type parasites with an IC_50_ = 0.21 μM. A significant drop in inhibitor potency was observed with the GNF7686-resistant *T*. *cruzi* epimastigotes (oxygen consumption IC_50_ = 5.2 μM). As seen for growth inhibition, the results on respiration inhibition distinguish GNF7686 and antimycin A from the Q_P_ site inhibitors (Fig 4B).

We then asked whether GNF7686 inhibits *T*. *cruzi* cytochrome *bc*_*1*_ (complex III) directly (Fig 4C). Epimastigotes were permeabilized with digitonin, and KCN (complex IV inhibitor) was added to the permeabilized cells to block electron conductance by the parasite ETC. The reaction was then initiated by adding stoichiometric quantities of decylubiquinol (an electron donor for complex III) and oxidized yeast cytochrome *c* (an electron acceptor), and the catalytic activity of complex III was monitored through accumulation of reduced yeast cytochrome *c*. A control reaction with antimycin A confirmed earlier observations with intact epimastigotes (Table 1). Antimycin A potently blocked reduction of cytochrome *c* in the reaction with wild-type permeabilized epimastigotes, but a dramatic loss of inhibitor potency was observed (~100-fold) when GNF7686-resistant permeabilized parasites were used (Fig 4C). In a similar fashion, GNF7686 inhibited wild-type complex III activity with an IC_50_ of 0.40 μM, but a 20-fold loss of potency was observed with the mutant complex III (Fig 4C). These observations validate GNF7686 as a complex III inhibitor that likely targets the Q_N_ site of the *T*. *cruzi* cytochrome *b*.

### GNF7686 is highly selective for *T*. *cruzi* and does not inhibit mammalian respiration

The inhibitory effect of GNF7686 on mammalian cytochrome *b* function was assessed through monitoring oxygen consumption by mitochondria isolated from rat skeletal muscle cells. In the control reaction, antimycin A showed a potent inhibition (IC_50_ = 0.81 μM) of mitochondrial respiration (Fig 4D). In a parallel experiment, GNF7686 did not show any effect on oxygen consumption up to 25 μM concentration (Fig 4D). This observation validates GNF7686 as a highly selective inhibitor of the *T*. *cruzi* cytochrome *b* and a promising starting point for Chagas disease drug discovery.

We also examined effect of GNF7686 on cytochrome *b* in the malaria parasite, *P*. *falciparum*, and in yeast *S*. *cerevisiae*, the latter being used as a surrogate for pathogenic *Pneumocystis jirovecii*, a causative agent of a pneumocystis pneumonia [59]. Cytochrome *b* is a validated drug target in both organisms and atovaquone, an inhibitor of cytochrome *b*, is a clinical treatment for these diseases.

For the *P*. *falciparum* studies, all inhibitors were tested on two parasite lines—D10attB and yDHODH-D10attB (Table 2). In the wild-type D10attB line, the parasite *de novo* pyrimidine biosynthesis is dependent on a type 2 dihydroorotate dehydrogenase (*Pf*DHODH) and requires a functional *P*. *falciparum* ETC, including cytochrome *b*, downstream from *Pf*DHODH [26, 27]. In contrast, the yDHODH-D10attB cell line is modified with a type 1A dihydroorotate dehydrogenase from *S*. *cerevisiae* (yDHODH), which is cytosolic and utilizes fumarate as the terminal electron acceptor [25–27]. The assay was validated with antimycin A, which blocked growth of the D10attB line (EC_50_ = 0.7 μM), but was inactive on the yDHODH-D10attB parasite line (EC_50_ > 12.5 μM). Myxothiazol and strobilurin also showed a similar preferential activity on the D10attB line, whereas GNF7686 did not inhibit growth of either *P*. *falciparum* cell line. This result suggests that GNF7686 is not active on *P*. *falciparum* cytochrome *b*.

A similar, growth inhibition-based assessment of GNF7686 effect on the ETC function was also performed on *S*. *cerevisiae* (Table 2). Growth inhibition of a wild-type *S*. *cerevisiae* strain by compounds in two different media was monitored. The first medium contained glucose as the sole carbon source, which allows growth of yeast cells that lack a functional ETC [60]. In the second medium, glucose was replaced with glycerol, a non-fermentable carbon source. Under the latter condition, yeast growth is dependent on cellular respiration and functional cytochrome *b* [60]. All prototypic cytochrome *b* inhibitors used in this report potently inhibited yeast growth on medium with glycerol, but were inactive when yeast grew in medium with glucose. Following a similar pattern, GNF7686 weakly inhibited growth of yeast in glycerol medium (EC_50_ = 5.0 μM), but did not affect yeast growing in the medium with glucose.

In summary, GNF7686 selectively inhibits *T*. *cruzi* cytochrome *b* and does not affect respiration of mammalian mitochondria nor does it significantly inhibit respiration-dependent growth of *P*. *falciparum* and *S*. *cerevisiae*.

## Discussion

We have shown that cytochrome *b* is a possible target for new drug discovery efforts aimed at treating kinetoplastid diseases. The importance of this finding is underlined by the paucity of drug targets for these diseases. For Chagas disease, only sterol 14α-demethylase (CYP51) and cruzain have been explored in depth as possible targets. However, low parasitological cure rates that were observed (20–30%) during clinical testing of anti-fungal drugs targeting sterol 14α-demethylase (posaconazole and ravuconazole prodrug E1224) in Chagas disease patients have lessened enthusiasm for further work on repurposed CYP51 inhibitors as single agents [61, 62]. Additional clinical evaluation of this class of drugs partnered with benznidazole for combination treatments is still planned. It is also important to note that the failure of posaconazole and E1224 in phase 2 trials have been attributed to insufficient drug exposure or dosing duration [14], and work on *T*. *cruzi*-specific CYP51 inhibitors that could enter clinical development in the future is also ongoing [63, 64]. Finally, the anti-cancer drug BEZ235 has activity across kinetoplastid parasites, but it requires additional optimization (improvement of therapeutic index) before becoming a preclinical candidate [65]. Given this sparse landscape, new chemical starts and new drug targets are urgently needed to anchor drug discovery efforts for the kinetoplastid diseases.

Many groups have resorted to a ‘pre-genomic’ approach to drug discovery, in which compounds are screened to identify inhibitors of pathogen growth, without regard to mechanism of action. While this approach typically provides large number of chemical starting points and broad hit diversity, a lack of information on mechanism of action creates additional risk in chemical optimization, and in predicting possible toxicity liabilities. Next generation sequencing of evolved resistant pathogens has been used successfully to identify resistance mechanisms and, in many cases, target mechanisms, for several pathogens. In our own program, we have identified targets for malaria and tuberculosis [66, 67]. However, the approach has not been reduced to practice in kinetoplastid drug discovery until this study.

Several features of the results in this study bode well for future application of the approach. First, the number of mutations associated with the emergence of drug resistance was relatively low (five point mutations), which simplified subsequent target prediction and validation. Second, we were able to generate resistance in *T*. *cruzi* epimastigotes despite a relatively long doubling time and stable genome. Selection process required almost a year of drug pressure in this study but was ultimately successful. Finally, we found a mutation in a gene that is a ‘plausible’ drug target, where plausibility is supported by essentiality of the mutated gene in other cellular systems, or precedence from drugs targeting homologous targets in other organisms.

Drugs targeting cytochrome *b* are in clinical use for treatment of malaria and fungal pneumonia, and cytochrome *b* was also reported as a promising target for treatment of tuberculosis. The current study extends utility of cytochrome *b* as a drug target also to Chagas disease, and possibly leishmaniasis. Various structurally different cytochrome *b* inhibitors showed patterns of growth and biochemical inhibition that consistently confirmed that functional cytochrome *b* is essential for *T*. *cruzi* propagation. Based on the presented validation data, *T*. *cruzi* growth can be inhibited through targeting either Q_N_ or Q_P_ cytochrome *b* site. GNF7686 represents a new cytochrome *b* inhibitor, likely targeting the Q_N_ site_,_ which has high selectivity for *T*. *cruzi* and does not show any effect on respiration of mammalian mitochondria. Crystal structures of cytochrome *b* from several sources (bovine, chicken, yeast, *Rhodobacter*, *Paracoccus*) were previously published [68, 69]. This opens opportunities for rational drug design based on homology modeling of *T*. *cruzi* cytochrome *b* structure. Finally, a counter-screen assay to measure inhibitory activity on the cognate human enzyme (as described here) can be used to guide chemical optimization away from host toxicity. While GNF7686 appears to be a promising starting point for kinetoplastid drug discovery, it does not inhibit the growth of *P*. *falciparum* or *S*. *cerevisiae*, and thus may not be a suitable starting point for anti-malaria or anti-fungal drug discovery. Further *in vivo* characterization of GNF7686 revealed that it has poor pharmacokinetic properties, including low oral bioavailability (F = 6%) and high *in vivo* mouse clearance (53 mL * min^-1^ * kg^-1^); thus, extension of the current studies to an *in vivo* model of Chagas disease will require identification of a GNF7686 analogue that has improved pharmacokinetic profile.

In summary, we have established an approach for identification of molecular targets of *T*. *cruzi* growth inhibitors that enables transition to target-based drug discovery for compounds with previously unknown mechanism of action. The first application of this approach resulted in identification of a highly selective inhibitor of *T*. *cruzi* cytochrome *b*, GNF7686, which can serve as an excellent starting point for discovery of new drugs for Chagas disease and leishmaniasis.
